# Arabidopsis RanBP2-Type Zinc Finger Proteins Related to Chloroplast RNA Editing Factor OZ1

**DOI:** 10.3390/plants9030307

**Published:** 2020-03-01

**Authors:** Andrew B. Gipson, Ludovic Giloteaux, Maureen R. Hanson, Stephane Bentolila

**Affiliations:** Department of Molecular Biology and Genetics, Cornell University, Ithaca, NY 14853, USA; abg226@cornell.edu (A.B.G.); lg349@cornell.edu (L.G.); sb46@cornell.edu (S.B.)

**Keywords:** RanBP2, zinc finger, RNA editing, RNA processing, RNA-binding proteins

## Abstract

OZ1, an RNA editing factor that controls the editing of 14 cytidine targets in Arabidopsis chloroplasts, contains two RanBP2-type zinc finger (Znf) domains. The RanBP2 Znf is a C4-type member of the broader zinc finger family with unique functions and an unusually diverse distribution in plants. The domain can mediate interactions with proteins or RNA and appears in protein types such as proteases, RNA editing factors, and chromatin modifiers; however, few characterized Arabidopsis proteins containing RanBP2 Znfs have been studied specifically with the domain in mind. In humans, RanBP2 Znf-containing proteins are involved in RNA splicing, transport, or transcription initiation. We present a phylogenetic overview of Arabidopsis RanBP2 Znf proteins and the functional niches that these proteins occupy in plants. OZ1 and its four-member family represent a branch of this family with major impact on the RNA biology of chloroplasts and mitochondria in Arabidopsis. We discuss what is known about other plant proteins carrying the RanBP2 Znf domain and point out how phylogenetic information can provide clues to functions of uncharacterized Znf proteins.

## 1. Introduction

The zinc finger domain is most well-known as a DNA-binding domain present in numerous transcription factors, but it is in fact a superfamily with several sub-families characterized by various structural and functional differences. One zinc finger (“Znf”) family that breaks with the classical DNA-binding function is the RanBP2 family. The namesake of the RanBP2 zinc finger family is Ran Binding Protein 2, a human protein whose cluster of eight zinc fingers participate in protein binding with the nuclear export factor exportin-1 [1]. There are other proteins associated with the nuclear pore whose RanBP2-Znf domains participate in protein–protein interactions, such as Nup153 [2]. Particularly relevant to this review is ZRANB2, a human protein that is known to be a component of the nuclear spliceosome and has influence over alternative splicing in several transcripts related to cell migration (e.g., SPATA13) and chromatin remodeling (e.g., SMARCC2), among other functions [3,4]. The RanBP2 Znf domains of ZRANB2 have a high affinity for the RNA sequence GGU [5], which is the core sequence in the 5′ splice site of the majority of spliced transcripts in both humans [6] and plants [7]. ZRANB2 also binds to and stabilizes a long non-coding RNA, SNHG20, which is involved in reducing the formation of nutrient-obtaining structures in glioma tumors [8]. ZRANB2 is not alone in this subclass of RNA-binding RanBP2 proteins; seven human proteins, including ZRANB2, share highly similar RanBP2-Znf domains, and almost all are involved in RNA splicing, transport, or transcription initiation [9].

**Figure 1:**
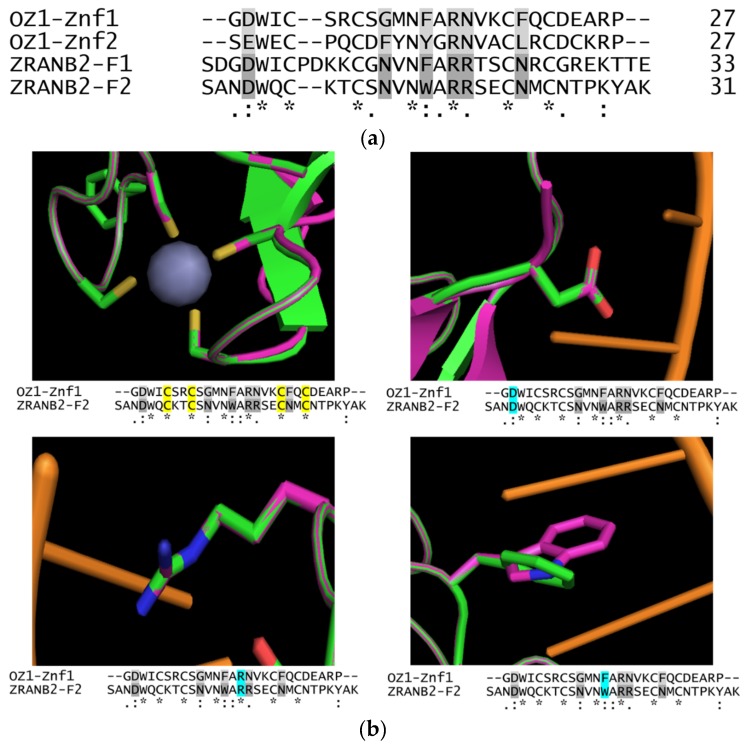
Model comparison of ZRANB2-Znf2 crystal structure with predicted structure of OZ1-Znf1. (**a**) Sequence alignment of the zinc finger domains of human ZRANB2 (“ZRANB2-F1 and ZRANB2-F2”) and of OZ1 (“OZ1-Znf1 and OZ1-Znf2”). Asterisks: conserved RanBP2 Znf residues; double dots: chemically similar residues; gray shading: putative RNA-binding residues. (**b**) Overlaid models of ZRANB2-F2 w/RNA target crystal structure and OZ1-Znf1 predicted structure comparing ZRANB2-F2 RNA-binding residues with OZ1-Znf1 residues in the same position. Blue highlighting in sequence alignment corresponds to the residue in the image. Green: OZ1 Znf-1; purple: ZRANB2; orange: RNA from ZRANB2 crystal structure; gray sphere: Zn^2+^ ion. Upper left: Zn^2+^-coordinating Cys residues; upper right: Glu residue of ZRANB2-F2 hydrogen bonds with guanine through water molecule; bottom left: Arg residue of ZRANB2-F2 hydrogen bonds with guanine at two positions; bottom right: Trp residue of ZRANB2-F2 base-stacks between adjacent guanines. Protein Database (PDB) ID for ZRANB2 structure: 3g9y. OZ1 Znf-1 modeled with Phyre2 [11] using ZRANB2-F2 structure as a base (Phyre2 homology confidence = 99.2%; percent ID = 36).

Our group became interested in the RanBP2 family of Znfs after our discovery of OZ1, an essential Arabidopsis chloroplast RNA-editing factor that has two tandem RanBP2-type Znfs [10]. The sequences of the OZ1 Znfs are remarkably similar to those of ZRANB2 (Figure 1a). The consensus sequence of the RanBP2 Znf is R/K-X-G-D-W-X-C-X(2,4)-C-X(3)-N-X(6)-C-X(2)-C-X(3)-R/K (30 residues); the four Cys residues distinguish this Znf from some other Znf families and are required for coordination of a Zn^2+^ necessary for the structural fold of the domain. Predictive structure modeling of an OZ1 Znf, using the solved structure of the second Znf of ZRANB2 as a starting point and comparison, shows several residues of OZ1 in the same position as the RNA-binding residues of ZRANB2 (Figure 1b), suggesting that this organellar RNA editing factor may have similar RNA-binding properties. As will be discussed later, there is already evidence that other Arabidopsis proteins with the RanBP2 Znf domain perform RNA binding.

In the remainder of this paper, we will discuss a number of other RanBP2 Znf proteins found in Arabidopsis, comparing their sequences and localization along with uncharacterized RanBP2 Znf proteins, and then highlight the need to further study this family in plants.

## 2. Subcellular Location of RanBP2 Zinc Fingers in Arabidopsis

Arabidopsis proteins possessing RanBP2 Znfs (InterPro ID: IPR001876) were identified by querying the Interpro protein database (version 77.0) [12], and redundant entries were identified with CD-HIT [13], narrowing down the selection to 26 proteins (Table 1). In considering what proportion of these proteins may have organellar localization, a mix of experimental and algorithmic prediction results from the SUBA4 protein localization database indicates that 3.9% of Arabidopsis proteins are mitochondrial, and 4.4% are plastidial, for a total of 8.3% of proteins being in one of those organelles [14]. Analysis of the proteins in this study show that the majority of Arabidopsis RanBP2 Znf proteins are nuclear-localized (~58%), with a portion being predicted or demonstrated to be in plastids and/or mitochondria (23%), largely due to the OZ family (Table 1).

Comparison of the results from TargetP 2.0 [15] and Predotar [16] localization algorithms against the SUBA4 database, which compiles information from TargetP and Predotar along with other algorithms and empirical evidence, shows that localization prediction is not always accurate (Table 1). OZ1, for instance is rather weakly predicted to be plastid-localized by TargetP and strongly predicted to be non-organellar by Predotar, but all localization experiments have shown it to be exclusively in the plastid [10]. RBL14 is an example of a protein with controversial localization; the primary Arabidopsis algorithm TargetP predicts mitochondria, and SUBA4 strongly suggests plasma membrane localization based on a large-scale fluorescent labeling experiment [17], but the image data from the study do not exclude mitochondrial localization in addition to plasma membrane localization. In any case, empirical studies focused on the protein in question are required for confident statements about its location.

## 3. Phylogenetic Analysis of Arabidopsis Proteins Carrying RanB2 Zinc Fingers

**Figure 2:**
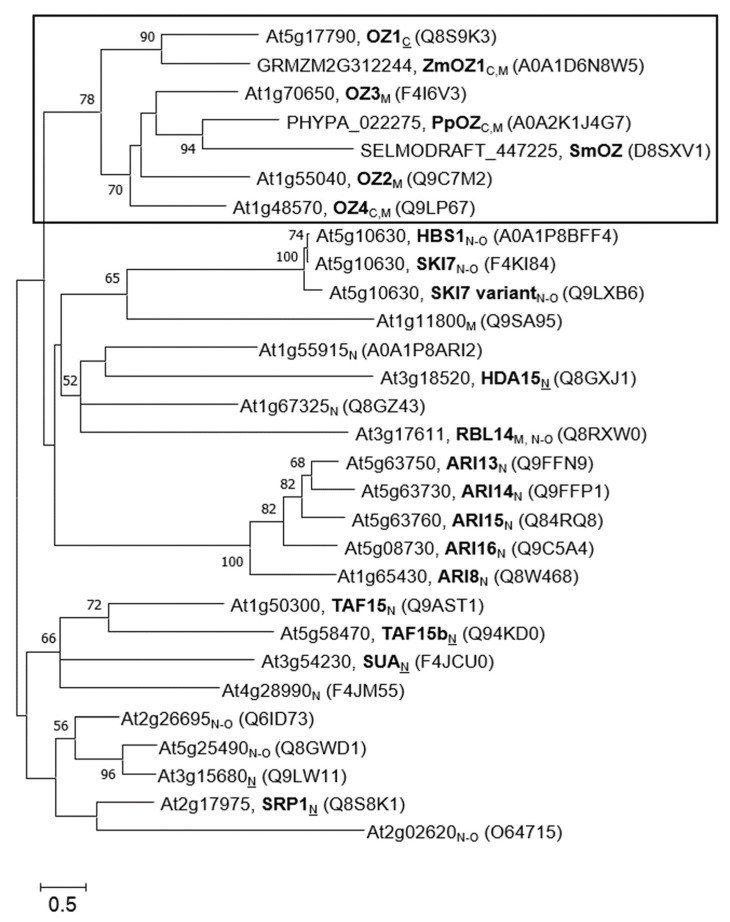
Phylogenetic tree of full-length proteins containing RanBP2 zinc finger domains in Arabidopsis. RanBP2 Znf protein sequences from *Zea mays* (ZmOZ1), *Physcomitrella patens* (PpOZ), and *Selaginella moellendorffii* (SmOZ) are included for evolutionary context. Entries list the TAIR accession number (or the species-specific accession number), the protein name in bold, and the UniProt ID in parentheses. Subscripts indicate localization: C = chloroplast, M = mitochondria, N = nucleus, N-O = non-organellar; non-underlined letter indicates predicted localization, underlined letters indicate experimentally confirmed localization. The evolutionary relationships were examined by sequence alignment using MUSCLE [20] and analysis was conducted in MEGA X [24]. A phylogenetic tree was inferred by using the Maximum Likelihood method and the Whelan and Goldman model [21]. The tree is drawn to scale, with branch lengths measured in the number of substitutions per site. Percentages of 500 bootstrap resampling that supported the branching orders in each analysis are presented above or near the relevant nodes and are shown for branches with more than 50% bootstrap support. This analysis involved 29 amino acid sequences with a total of 1288 positions considered in the final dataset.

Full-length protein sequences for phylogenetic analysis were obtained from UniProt [18] and verified against sequences in TAIR [19]. MUSCLE alignment [20] was used to align protein sequences, and phylogenetic relationships were drawn with the Maximum Likelihood method as described in Whelan and Goldman [21]. Using the Znf sequence from OZ1 as a BLAST query, RanBP2 Znf proteins from model plants in other clades (*Zea mays*, the moss *Physcomitrella patens*, and the lycophyte *Selaginella moellendorffii*) were identified and included in the phylogenetic analysis to act as reference points for evolutionary speculation. The most noticeable feature of the phylogenetic tree is the grouping of OZ proteins into a single clade (Figure 2). In spite of the evolutionary distance between OZ proteins in Arabidopsis, *P. patens*, and *S. moellendorffii*, their grouping may imply similar functions of these proteins, possibly in organellar RNA processing like OZ1 [10]. Another notable clade is the Ariadne (ARI) family, E3-type ubiquitin ligases with unique C-terminal RanBP2 Znfs. This protein family is discussed in detail in Section 4.8, but it is worth mentioning here because this group is like the OZ group in that all members have at least been named and commented upon in the literature, if not individually characterized [22,23]. Conversely, the remaining nodes on the tree in Figure 2 include at least one wholly uncharacterized protein. Each of these RanBP2 Znf proteins can be seen as a potential new splicing factor, chromatin modifier, protease, or RNA processing factor, based on its association with RanBP2 Znf proteins whose functions have been established.

**Figure 3:**
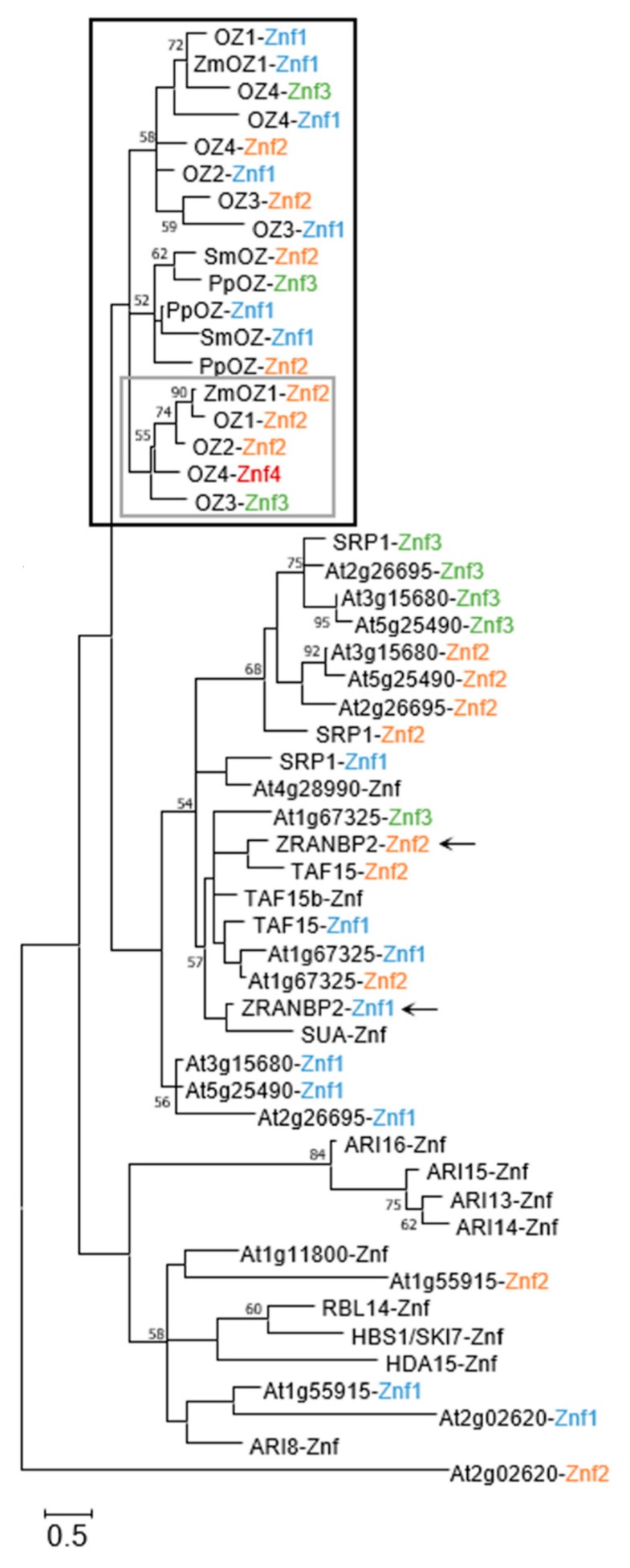
Phylogenetic tree of Arabidopsis RanBP2 Zinc Finger domains. ZRANB2 (marked with arrows) is a human RanBP2 Znf protein included for comparison. Black box: OZ clade; gray box: clade of C-terminal OZ Znf domains. Text color based on the order of the zinc finger domain in the protein starting from the N-terminus: blue = first Znf, orange = second Znf, green = third Znf, red = fourth Znf; Znfs from proteins with only one such domain are colored black. RanBP2 Znf domain sequences were aligned using MUSCLE [20], and evolutionary analysis was conducted in MEGA X [24]. The tree was inferred by using the Maximum Likelihood method and the Whelan and Goldman model [21]. The tree is drawn to scale, with branch lengths measured in the number of substitutions per site. Percentages of 500 bootstrap resampling that supported the branching orders in each analysis are presented above or near the relevant nodes and are shown for branches with more than 50% bootstrap support. This analysis involved 53 amino acid sequences with a total of 38 positions considered in the final dataset.

The nature of protein domains as tools within different protein “toolboxes” led us to analyze the RanBP2 Znf domains themselves and how they interrelate, both for insights on their function as well as how they are related evolutionarily. The RanBP2 Znf has a unique signature (see consensus sequence in the Introduction) and is well-conserved across its different iterations, but examination of their number and sequence differences can give clues as to their function within the full-length protein. Sequences of individual RanBP2 Znf domains were extracted from UniProt domain designations, then the alignment and tree were assembled as described above. The Znf domains of the human splicing factor ZRANB2 were included as a reference point because their function and structure have been exhaustively determined and can provide insight into how the neighboring Znfs from Arabidopsis may function. Looking at this domain-based tree, we see again that the OZ family Znfs group together (Figure 3, black box). All OZ proteins have at least two RanBP2 Znf domains, and, interestingly, we see that the Znfs in the most C-terminal position group together within the OZ clade (Figure 3, gray box). Preliminary data indicate that the Znf in this position has the most impact on OZ1 editing function [25], so the conservation we observe may be indicative of selection on Znfs in that position related to their functional relevance.

**Figure 4:**
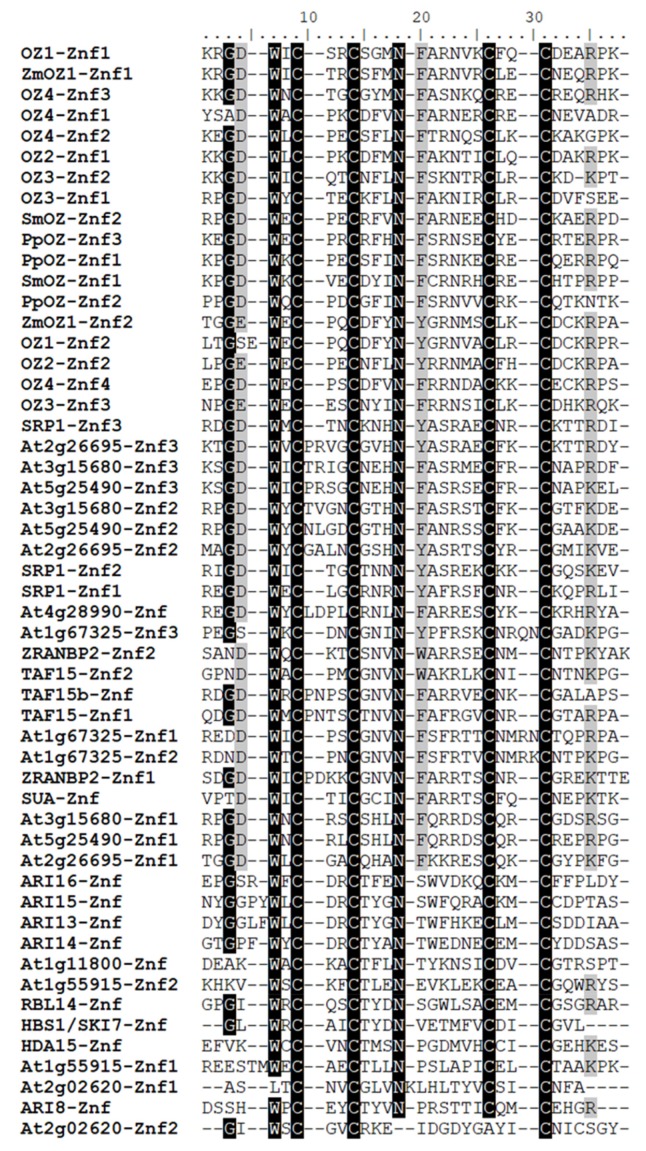
Alignment of RanBP2 Znf domains in Arabidopsis. MUSCLE alignment that was used to generate tree in Figure 3. Shading applied to positions with either 70% identical amino acids (black) or similar amino acids (gray).

Looking at other groups, we see that the divergence of the RanBP2 Znf domain does not always correspond to what is observed in the full-length protein phylogeny. For instance, the Znf of the ubiquitin ligase ARI8 is not in a clade with the other ARI proteins, reflecting its genetic distance from those proteins in spite of the similarity between the full-length ARI proteins (see Section 4.8). The second Znf of At2g02620 is particularly unusual, as it has lost both the well-conserved asparagine and the third putatively Zn^2+^-coordinating cysteine residue (Figure 4). As in the full-length phylogeny, looking at the closest characterized relative to a Znf from an uncharacterized protein in this tree can provide hypotheses as to their function. Znfs from the uncharacterized protein At1g67325 all group with known RNA-binding Znfs from ZRANB2 as well as putatively RNA-binding Znfs from TAF15, TAF15b, and SUA, strongly suggesting that At1g67325 could be an RNA-binding protein itself. We will explore more of these possibilities in Section 5.

## 4. RanBP2 Proteins with Known Functions

### 4.1. OZ1 and Family

**Figure 5:**
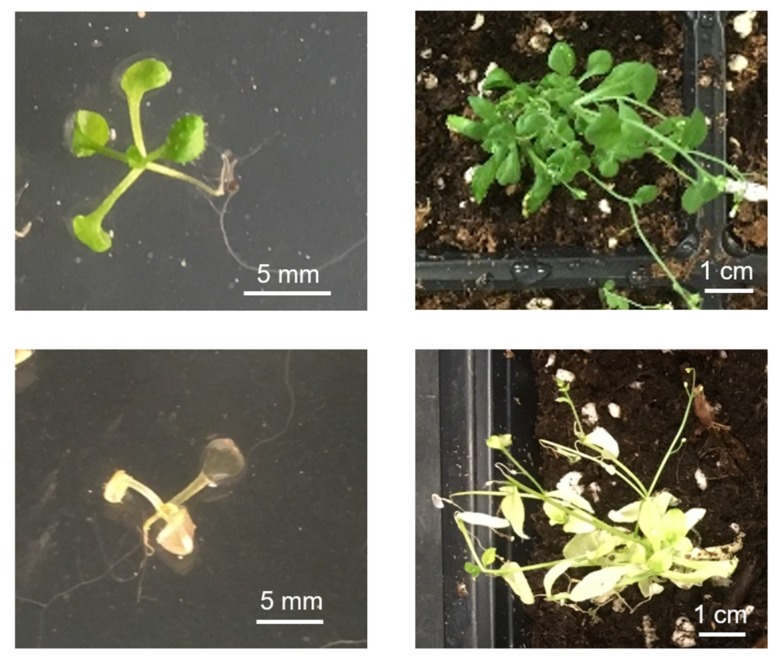
Wild-type (WT) Arabidopsis: 10 days old (**top left**) and 2 months old (**top right**); *oz1-1* homozygous mutant: 11 days old (**bottom left**) and 2 months old (**bottom right**).

The Organellar Zinc Finger (OZ) family was named as a result of the identification of OZ1 as a chloroplast RNA editing factor [10]. Although OZ1 had been described before as a variegated mutant of unknown function named VAR3, its importance for chloroplast RNA editing was not detected at that time [26]. In the *oz1-1* insertional mutant used in Sun et al. (2015), the absence of OZ1 leads to delayed chloroplast development and decreased germination rates (Figure 5), but not variegation. The *var3* mutant is a *Ds* mutant in Landsberg *erecta*, while the *oz1-1* line is a T-DNA mutant in Columbia background; the reason for the discrepancy in phenotype is not known. The small protein family that includes OZ1, which has two Znfs, is also comprised of OZ2 (two Znfs), OZ3 (three Znfs) and OZ4 (four Znfs). These proteins have Znf domains well-conserved among them as well as a heretofore uncharacterized and uncatalogued N-terminal set of motifs unique to the family. At least two of the OZ proteins have been identified to be crucial for RNA processing in their respective organelles, and the remaining two are observed to be located in plastids and/or mitochondria as well [10,25].

It is not yet known whether the OZ1 Znf domains are capable of binding ssRNA like the ZRANB2 Znf domains. Recent experiments, however, indicate that the Znf domains of OZ1 are necessary for its function in chloroplast RNA editing. Full-length OZ1 is known to bind to other chloroplast RNA editing factors; indeed, the protein was originally discovered due to its interaction with the editing factor ORRM1 [10]. However, the RanBP2 Znfs are unlikely to be the protein-interacting variety of Znfs because a region of OZ1 other than the Znfs was observed to participate in those interactions [25].

### 4.2. RHOMBOID-Like Protein 14 (RBL14)

Rhomboids are membrane-anchored serine proteases chiefly involved in the proteolytic maturation, activation, or inhibition of protein targets [27]. RBL14 (named RBL10 in some studies) is considered to be in the “secretase-B” class, characterized by having six trans-membrane domain helices. The closest well-characterized homolog of RBL14 is the human protein RHBDL4 (alternative name RHBDD1, UniProt acc. Q8TEB9), which is involved in proteolytic maturation of ER proteins, among other functions [28]. Based on the sequence similarity to ER-localized RHBDL4, Lemberg and Freeman [29] state that secretase-B rhomboids are not mitochondrial, contrary to prediction by algorithms (Table 1). However, AtRBL14 has only 44.3% sequence similarity and only 31.2% sequence identity with RHBDL4 [29]. Importantly, RHBDL4 does NOT have a Znf domain, but the rice ortholog of AtRBL14 does [30], so either the RanBP2 Znf became incorporated into this protein after the plant ancestor split from the animal/fungal ancestor, or the Znf was lost in the other lineage. Based on a loose prediction of RBL14’s structure [29], the C-terminally positioned Znf of RBL14 could serve to interact with soluble domains of the protein targets unique to RBL14. There is precedent for Arabidopsis homologs of characterized RBL proteins in other taxa to have different substrate specificity; another Arabidopsis RBL, RBL12, does not process yeast targets of the corresponding yeast rhomboid, unlike the corresponding human rhomboid [31], and AtRBL1 could not cleave substrates of its *Drosophila* homolog RHO-1 [32]. This implies that the substrates of AtRBL14 may differ significantly from those of the homologous yeast, *Drosophila*, and human proteins.

Using Genevestigator, Knopf and Adam [27] determined that RBL14 (named RBL10 in their review) is expressed consistently across plant tissues and across development, but they also found that RBL14 may be upregulated in response to heat shock. An attempt to identify the location of the protein through green fluorescent protein labeling was inconclusive [31]. The published images of the transfected protoplasts labeled with MitoTracker-orange do not rule out mitochondrial localization. The contribution of the Znf to the putative function of RBL14 has not been assessed, but as noted earlier, it might facilitate interaction with proteolytic targets.

### 4.3. Histone Deacetylase 15 (HDA15)

HDA15 was first found by querying the Arabidopsis genome sequence with histone deacetylase protein sequences from yeast, *Drosophila*, human, maize, and mouse [33]. It is considered to be part of the Class II group of histone deacetylases [33]. HDA15 is expressed highly in the stems throughout the life of the plant, and it may undergo partial export from the nucleus under dark conditions, reentering the nucleus when the protoplasts are returned to light [34].

HDA15 is involved in the repression of chlorophyll synthesis in the dark that is mediated by PIF3, a transcription factor [35]. PIF3 binds to genes related to photosynthetic activity and represses them through the histone deacetylase activity of its binding partner, HDA15. PIF3 recruits HDA15 to the promoter regions of target genes by binding to the G-box sequence. HDA15 is one of the few proteins we reviewed for which there is direct evidence for the function of the RanBP2 Znf: a Znf-containing truncation of HDA15 binds to PIF3 in vitro. HDA15 binds to the transcriptional repressor PIF3 in the absence of light, and HDA15 is confirmed to be nuclear-localized [35]. The location of HDA15 did not change in response to light treatment in the Liu et al. study [35], so the putative cytoplasmic shuttling proposed by Alinsug et al. [34] may not be related to its function in repression of genes involved in photosynthesis. HDA15 levels are not changed by light; it is the phosphorylation and degradation of PIF3 that reduces HDA15 deacetylation of histones at the affected genes. This represents a classic case of transcriptional repression via recruitment of histone modifiers [35]. HDA15 has also been recently shown to be a direct repressor of plant thermal responsive genes at normal temperature [36].

Other transcription factors interact with the RanBP2 Znf domain of HDA15 to utilize it as a transcriptional repressor. For example, PIF1 opposes seed germination in the absence of light by repressing gibberellic acid-synthesizing genes [37], and MYB96 brings HDA15 to inhibit abscisic acid (ABA) signaling genes [38]. Nuclear Factor-YC proteins (NF-YCs) are transcription factors that function in a group with other factors to induce chromatin modifications of genes controlling germination and light responses, and they also interact with HDA15 [39]. Unfortunately, these latter interaction studies did not use truncations or domain mutations of either protein, so we cannot draw any conclusions about which domains are required for interactions with NF-YC transcription factors. The most recent study of HDA15 found yet another protein interaction with HFR1, a transcription factor that works with HDA15 to repress the warm-temperature response [36]. That study detected protein–protein interactions between HFR1 and a Znf-containing truncation of HDA15 using yeast two-hybrid assays. Another truncation containing the HDA15 Znf did not interact with HFR1; that construct removed the first two residues of the Znf.

### 4.4. TATA-Binding Protein-Associated Factor 15 and 15b (TAF15 & TAF15b)

Using a consensus sequence of TAF proteins built from humans, *Drosophila*, and yeast, Lago et al. [40] found, among other Arabidopsis TAF proteins, two that matched with human TAF15: TAF15 and TAF15b. Human TAF15 is most similar to Arabidopsis TAF15b, not to Arabidopsis TAF15. TAF15 was found to be a component of the general transcription factor TFIID. TAF15b was shown to be located in both the nucleus and cytosolic p-bodies [41]. TAF15b has an RGG-rich region in the C-terminus that may direct it to the p-bodies, based on similar domains confirmed to localize human TAF15 to p-bodies [42]. TAF15b may control the stability or RNA processing of SNC1, a Toll-like receptor involved in plant immunity, in the p-bodies [41].

TAF15b suppresses flowering under conditions of vernalization [43]. TAF15b binds to Pol II in various states of phosphorylation, as shown by co-immunoprecipitation. TAF15b represses transcription elongation of Flowering Locus C (FLC), as determined by analysis of the phosphorylation state of Pol II in wild-type (WT) vs. *taf15b* mutants. Bertolotti et al. [44] found that the TAF15b-related human protein TAF15 (named hTAF_II_68 in that study) binds both RNA and single-stranded DNA; however, their tested truncations did not separate the RanBP2 Znf from the neighboring RNA Recognition Motif (RRM), so those binding activities cannot be assigned to one domain over the other.

### 4.5. Hsp70 Subfamily B Suppressor (HBS1)/Superkiller Protein 7 (SKI7)

The gene products of At5g10630 have splice isoforms predicted to have drastically different functions. The shorter splice form, named HBS1, may recognize and release stalled ribosomes, while the longer splice form, SKI7, likely incorporates into an RNA exosome [45]. These names and putative functions are based on their similarity to human and yeast homologs. The third predicted protein (UniProt ID: Q9LXB6; Table 1) is likely the product of SKI7 regulation via alternative splicing, producing an RNA targeted for nonsense-mediated decay. The Znf domain is present in all isoforms, as are a GTP-ase domain, Patch 4-like domain, and negatively charged N-terminal region [45]. The main feature separating SKI7 from HBS1 is the presence of a 66-amino acid region called the SKI7-like motif, which is known to be the domain that tethers SKI7 to the RNA exosome [46].

A recent study showed that HBS1 contributes to degradation of 5′ cleavage products from RNA silencing as well as releasing RNA from stalled ribosomes [47]. Aside from these “non-stop” RNA decay processes, carried out on RNAs lacking stop codons, HBS1 is also necessary for “no-go” decay, where transcripts are degraded when the ribosome is stalled [48]. It does this by entering the ribosome A-site along with two other proteins, Pelota2 and SKI2, and exposing the RNA to endonucleolytic cleavage. SKI7 associates with a completely different set of proteins to participate in RNA degradation; it is not an endonuclease itself, but at least one other protein in the SKI-exosome complex does have endonucleolytic activity [49]. The presence of a RanBP2 Znf in both isoforms could be an example of a protein-binding function for this Znf domain; the evidence from the reviewed studies point to the Znf being used to bind protein partners in these RNA-processing complexes. Its similarity to the Znf of HDA15 (Figure 3), which binds to a variety of different protein partners, further supports this possibility.

### 4.6. Suppressor of ABI3-5 (SUA)

SUA is a splicing factor containing two RRM domains, a RanBP2 Znf, an octamer repeat region, and a glycine-rich domain [50]. SUA prevents the splicing of an intron in ABI3, a transcription factor in the ABA signaling pathway [51], which is responsible for seed maturation; if spliced, this intron produces an ABI3 truncation in WT that does not function. *abi3-5* mutant plants are insensitive to ABA and produce green seeds with low viability, but these mutants produce a functional form of ABI3 when that intron is spliced in *abi3-5 sua-1* double mutants. Expression of SUA: GFP under the control of the SUA promoter in *abi3-5 sua-1* double knockout plants caused them to revert to the green seed phenotype of the *abi3-5* mutant. SUA is concentrated in the nucleus and binds with the spliceosomal factor U2AF65, suggesting that SUA is directly integrated with the spliceosome [50].

SNC4, a receptor-like kinase involved in plant immunity, retains an intron in *sua* mutants. SUA is also needed for splicing of CERK1, another receptor-like kinase, and lack of CERK1 splicing in *sua* mutants degrades their ability to resist pathogens [52]. Both the Sugliani et al. [50] and Zhang et al. [52] studies implicate SUA as a splicing factor. It is possible that the Znf in SUA binds to either the 5′ or 3′ splice site in a manner similar to ZRANB2.

### 4.7. Stress Associated RNA-Binding Protein 1 (SRP1)

First discovered for its homology to a stress response RNA-binding protein in rice, Stress Associated RNA-binding protein 1 (SRP1) has been recently described as a post-transcriptional regulatory protein containing three RanBP2 Znf domains [53]. Its specific target is the 3′ UTR of *ABI2*, a transcript coding for a phosphatase involved in regulation of the ABA signaling pathway [51]. The expression of known ABA signaling genes was altered in an opposite manner in *srp1* knockouts vs. SRP1 overexpression plants (e.g., ABI2 expression increased in *srp1* mutants vs. WT but was decreased in SRP1 overexpression plants). Interestingly, ABI2 appears to be negatively regulated by SRP1 binding. It was shown to bind to 3′-UTR RNAs in vitro, potentially through the AUUUA sequences. This is the first Arabidopsis RanBP2 Znf-containing protein demonstrated to have RNA binding activity likely mediated by its Znf domain, as there are no other known motifs in SRP1.

### 4.8. Ariadne Family

The Ariadne (ARI) family is a group of putative E3-type ubiquitin ligases [22], the final enzymatic actor in a chain of reactions that ubiquitinate protein targets, marking them for degradation [54]. ARI proteins are typified by a pair of RING finger-type Znf domains, an unusual Znf typical of E3 ubiquitin ligases that binds two Zn^2+^ atoms with a 3-Cys, 1-His, 4-Cys motif [55]. The ARI proteins mentioned in this study (Table 1) are distinguished from the other Arabidopsis ARI proteins by the presence of a single RanBP2 Znf at the extreme C-terminal end. *ARI13*, *ARI14*, *ARI15*, and *ARI16* are close together on chromosome 5 of Arabidopsis and share similar gene architectures, which led Mladek et al. [22] to hypothesize that the cluster resulted from a series of gene duplication events; the subsequent diversification has led to notable functional differences. For example, ARI14 is regulated by a native siRNA, *KOKOPELLI* (*KPL*), in sperm cells to potentially counteract the ubiquitin-mediated protein degradation effected by ARI13 [56]. Due to ARI14’s mutations in the N-terminal RING finger domain, Ron et al. [56] speculate that it cannot act as an E3 ubiquitin ligase but can still bind to the ubiquitination complex in sperm cells, thus acting as a negative regulator of that activity.

The gene encoding ARI8 is located on a different chromosome and has an architecture much different from the ARI13/14/15/16 group. ARI8 has been demonstrated to perform ubiquitination in conjunction with a number of E2-type conjugating enzymes, as determined by in vitro assay [23]; these findings are the basis for considering the other ARI proteins as ubiquitin ligases. Despite the evolutionary distance of ARI8 from the ARI13/14/15/16 group, it has a C-terminal RanBP2 Znf. The Znf in ARI8 does differ in sequence from the other ARI Znfs (Figure 3 and Figure 4). The presence of the C-terminal RanBP2 Znf was not noted in the original description of the family [22], and no subsequent study has explored its function [23,56,57]. The close relationship of ARI RanBP2 Znf domains with that of HDA15, a protein which interacts with other proteins (Figure 3), suggests that they should be investigated further to determine whether they may mediate protein–protein interactions.

## 5. Uncharacterized RanBP2 Znf Proteins

After having surveyed the literature on Arabidopsis RanBP2 Znf proteins, we can now discuss the uncharacterized proteins and hypothesize as to their function by comparing with closely related characterized proteins and looking at the other domains present in the protein using NCBI Conserved Domain Search [58]. We can generally see that, in cases where the RanBP2 Znf is RNA-binding, it has a specific sequence to which it binds (e.g., ZRANB2 and SRP1). In contrast, several proteins containing RanBP2 Znfs apparently interact with multiple protein partners (e.g., HDA15 and RBL14). At1g55915 is on a node with HDA15 in the full-length tree (Figure 2) and possesses a WLM domain, which is a putative metalloprotease domain. If it does indeed function as a protease, perhaps the two Znf domains of At1g55915 conduct multiple protein interactions to facilitate proteolysis of multiple protein targets. At1g11800 is worth noting because it is predicted to be mitochondrially-localized. It contains a TDP2 domain, putatively a phosphodiesterase domain. This protein groups with HBS1/SKI7 (Figure 2), and so At1g11800 could have either ribosome-rescuing and/or RNA degradation duties in the mitochondrion. At4g28990 is a simple protein with just one Znf and no other detectable domains. It groups with TAF15 and TAF15b, but the Znf groups with SRP1-Znf1 (Figure 3). These associations suggest that At4g28990 may bind to RNA, but experimentation will be needed to learn the function of this protein and others. At1g67325, mentioned in Section 2, may have three RNA-binding Znfs (Figure 3), but the full-length protein loosely groups with HDA15 and RBL14, one of which is confirmed to have a protein-binding Znf. At2g02620 is a sister protein to SRP1 in the full-length phylogeny, but neither of its Znfs cluster with those of SRP1 (Figure 3); indeed, its first Znf is missing the signature tryptophan, and the second zinc finger has lost one of the four signature cysteines (Figure 4). This may be a protein for which the Znf is no longer necessary for its function. At5g25490, At2g26695, and At3g15680 are all small proteins with three RanBP2 Znfs. They are in the same clade as SRP1 (Figure 2) and may be additional RNA-binding proteins affecting the turnover and/or stability of transcripts. This group is the least studied of all the clades mentioned here and could represent a unique family of RNA-regulating factors.

## 6. Conclusions and Future Directions

Determining the function of these RanBP2 Znf domains in each protein can be a simple matter of producing truncated constructs of the protein that include and exclude the Znfs, or contain mutated Znfs, and testing those constructs for protein interaction and function in knockout mutants in vivo, as was done with HDA15. Expressing the domain itself to perform protein and RNA interaction experiments could help identify the function of Znf-containing proteins. Many of the proteins in this review are members of larger families, but they are distinguished from those families by their possession of a RanBP2 Znf. How does this domain contribute to the function of the protein in which it is located? During evolution, why has this domain become located in such a variety of proteins? What functional differences arise from having one versus three or four Znfs? These are questions that can only be answered by directly studying this versatile protein domain in multiple proteins.

## Figures and Tables

**Table 1:** RanBP2 Znf Proteins in Arabidopsis and Representative Model Plants.

Name	UniProt ID	TAIR ID	# of RanBP2 Znf Domains	Znf Function ^1^	SUBA Localization ^2^	TargetP ^2^	Predotar (v. 1.04, 2016) ^2^	Function ^1^
OZ1	Q8S9K3	At5g17790	2	N/A	Plastid (1)	Chloroplast (0.4837)	Non-Organellar (0.78)	*RNA editing*
OZ2	Q9C7M2	At1g55040	2	N/A	Plastid (1)	Mitochondrial (0.8581)	Plastid (0.41), Mitochondria (0.38)	N/A
OZ3	F4I6V3	At1g70650	3	N/A	Cytosol (0.579)	Mitochondrial (0.7018)	Mitochondria (0.71)	N/A
OZ4	Q9LP67	At1g48570	4	N/A	Plastid (0.993)	Mitochondrial (0.8274)	Non-Organellar (0.75)	N/A
RHOMBOID-like protein 14 (RBL14)	Q8RXW0	At3g17611	1	*Protein interaction*	Plasma membrane (1)	Mitochondria (0.9658)	Non-Organellar (0.72)	Serine protease
SKI7	F4KI84	At5g10630	1	*Protein interaction*	Cytosol (0.998)	Non-Organellar (0.9999)	Non-Organellar (0.99)	RNA degradation
SKI7-variant	Q9LXB6	At5g10630	1	*Protein interaction*	Cytosol (1)	Non-Organellar (0.9913)	Non-Organellar (0.97)	*RNA degradation*
HBS1	A0A1P8BFF4	At5g10630	1	*Protein interaction*	Cytosol (0.998)	Non-Organellar (0.9984)	Non-Organellar (0.99)	Stalled ribosome rescue
TBP-associated factor 15 (TAF15)	Q9AST1	At1g50300	2	*RNA binding*	Nucleus (1)	Non-Organellar (0.9998)	Non-Organellar (0.99)	Component of the general transcription factor TFIID
TBP-associated factor 15B (TAF15b)	Q94KD0	At5g58470	1	*RNA binding*	Nucleus (1)	Non-Organellar (0.9999)	Non-Organellar (0.99)	PolII inhibition
SUPPRESSOR OF ABI3-5 (SUA)	F4JCU0	At3g54230	1	*RNA binding*	Nucleus (1)	Non-Organellar (1)	Non-Organellar (0.99)	Splicing Factor
Histone Deacetylase 15 (HDA15)	Q8GXJ1	At3g18520	1	*Protein interaction*	Nucleus (1)	Non-Organellar (0.9977)	Non-Organellar (0.99)	Chromatin modification
ARI8	Q8W468	At1g65430	1	*Protein interaction*	Nucleus (1)	Non-Organellar (1)	Non-Organellar (0.99)	*Protein turnover/ubiquitination*
ARI13	Q9FFN9	At5g63750	1	*Protein interaction*	Nucleus (1)	Non-Organellar (0.9997)	Non-Organellar (0.99)	*Protein turnover/ubiquitination*
ARI14	Q9FFP1	At5g63730	1	*Protein interaction*	Nucleus (0.996)	Non-Organellar (1)	Non-Organellar (0.99)	*Protein turnover/ubiquitination*
ARI15	Q84RQ8	At5g63760	1	*Protein interaction*	Nucleus (0.997)	Non-Organellar (0.9967)	Non-Organellar (0.99)	*Protein turnover/ubiquitination*
ARI16	Q9C5A4	At5g08730	1	*Protein interaction*	Nucleus (0.999)	Non-Organellar (0.9999)	Non-Organellar (0.99)	*Protein turnover/ubiquitination*
Stress-associated RNA-binding Protein 1 (SRP1)	Q8S8K1	At2g17975	3	RNA binding	Nucleus (0.98)	Non-Organellar (0.9979)	Non-Organellar (0.99)	RNA turnover; binding of 3′-UTR of ABI2
-	Q9SA95	At1g11800	1	N/A	Mitochondria (0.999)	Mitochondria (0.7178)	Mitochondria (0.55)	N/A
-	Q8GWD1	At5g25490	3	N/A	Nucleus (0.54)	Non-Organellar (0.9895)	Non-Organellar (0.99)	N/A
-	F4JM55	At4g28990	1	N/A	Nucleus (1)	Non-Organellar (1)	Non-Organellar (0.99)	N/A
-	Q8GZ43	At1g67325	3	N/A	Nucleus (1)	Non-Organellar (0.9915)	Non-Organellar (0.98)	N/A
-	A0A1P8ARI2	At1g55915	2	N/A	Nucleus (0.999)	Non-Organellar (0.9174)	Non-Organellar (0.86)	N/A
-	O64715	At2g02620	2	N/A	Cytosol (0.542)	Non-Organellar (0.9994)	Non-Organellar (0.99)	N/A
-	Q6ID73	At2g26695	3	N/A	Plasma membrane (0.545)	Non-Organellar (0.7971)/Signal peptide (0.2023)	Non-Organellar (0.98)	N/A
-	Q9LW11	At3g15680	3	N/A	Nucleus (0.879)	Non-Organellar (0.9787)	Non-Organellar (0.99)	N/A
Pp_OZ	A0A2K1J4G7	PHYPA_022275	3	N/A	N/A	Mitochondria (0.4898)/Chloroplast (0.4733)	Plastid (0.88)	N/A
Sm_OZ	D8SXV1	SELMODRAFT_447225	2	N/A	N/A	Non-Organellar (0.6019)/Mitochondria (0.3215)	Non-Organellar (0.74)	N/A
Zm_OZ1	A0A1D6N8W5	GRMZM2G312244	2	N/A	N/A	Mitochondria (0.4279)/Chloroplast (0.3028)	ER (0.76), Plastid (0.41)	*RNA editing*

^1^ Italics indicate hypothetical function; non-italics indicate experimentally confirmed function. ^2^ Number in parentheses indicates the “confidence score” on a scale of 0 to 1; scores are determined independently within each database or algorithm.
